# Advanced Ovarian Dysgerminoma Infiltrating Both Ovaries and Uterus in a 7-Year-Old Girl

**DOI:** 10.1155/2014/910852

**Published:** 2014-02-23

**Authors:** Nexhmi Hyseni, Sadik Llullaku, Hysni Jashari, Kaltrina Zahiti, Fjolla Hyseni, Fisnik Kurshumliu, Lumturije Luci, Fehim Muqolli, Antigona Hasani

**Affiliations:** ^1^Department of Pediatric Surgery, University Clinical Centre, Laxha e Spitalit PN, 10000 Pristina, Kosovo; ^2^University Clinical Centre, Pathology Institute, 10000 Pristina, Kosovo; ^3^Department of Anesthesiology and Reanimation, University Clinical Centre, 10000 Pristina, Kosovo

## Abstract

*Introduction*. Ovarian dysgerminoma is a rare malignant ovarian germ cell tumor with its peak incidence in young women. Abdominal pain, abdominal distention, and the presence of a palpable mass are common symptoms at presentation. Depending on the FIGO stage at presentation the prognosis of dysgerminomas after surgical treatment, adjuvant chemotherapy, and radiotherapy is promising. *Case Presentation*. A 7-year-old girl was presented at our clinic with abdominal pain in all abdominal quadrants. Later the pain localized in the region of her right ovary. CT scan revealed a massive formation which was connected to her right ovary. *Conclusion*. Although malignant ovarian germ cell tumours are rare in children, physicians must always consider the possibility of MOGT-occurrences. The clinical symptoms might not be specific: abdominal pain, abdominal distention, nausea, and vomiting. In order to make a correct diagnosis the patients should undergo a complete clinical examination including radiological scans. Initial management is frequently surgery, followed by adjuvant chemotherapy and radiotherapy. Although disgerminoma is malignant tumor, the prognosis is promising.

## 1. Introduction

Dysgerminoma is a rare malignant ovarian germ cell tumor (MOGT) which is highly malignant and has its peak incidence in young women. Approximately one-third of all dysgerminomas show KIT mutations and these are associated with advanced stage at presentation [1]. Clinically the patients present with abdominal pain, abdominal distention, and presence of a palpable mass, reduced appetite, vomiting, and nausea as well as ovarian torsion [2, 3]. Conservative surgery, postoperative chemotherapy, and postoperative radiotherapy are effective therapeutic options. Fertility-preservation surgery is often possible [3] and the overall survival is 92.4% [4]. Preoperative elevation of tumor markers is significantly related to poor prognosis for progression-free survival (PFS) [4]. Dysgerminomas reveal in 28% of cases presence of lymph node metastasis, which is significantly associated with lower 5-year survival (82.8%) [5]. Older patients were more likely to be diagnosed at an advanced stage [6].

## 2. Case Presentation

**Figure 1:**
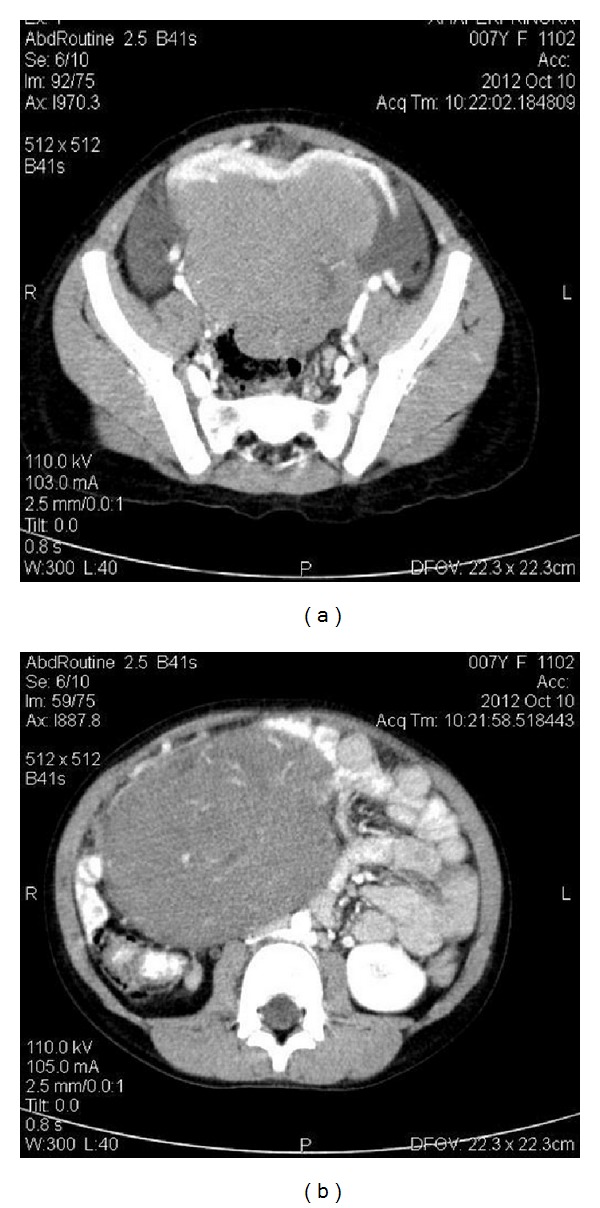
CT scan of the abdomen and pelvis revealed a massive, solid, and clearly bordered formation with the dimensions 12 × 8 × 15 cm. The suspicious formation showed a connection to the right ovary that was hyperdense and had an inhomogeneous appearance. Some hypodense tissue within the tumour mass was identified as probably “fatty tissue.”

**Figure 2:**
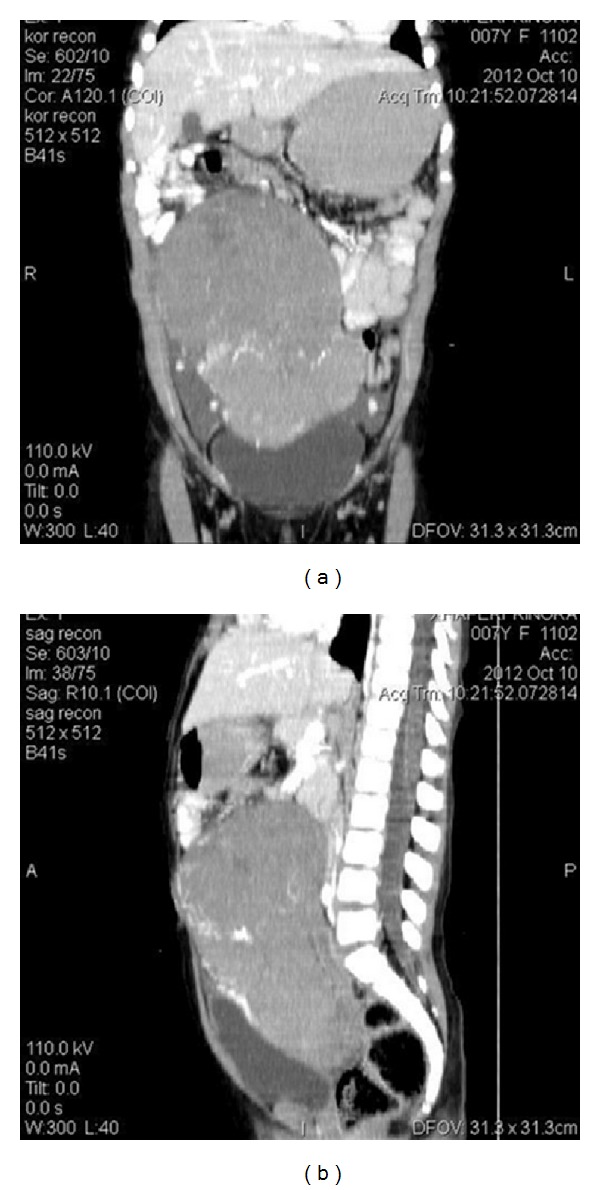
After an additional examination with intravenous contrast medium the tumour mass showed a raised vascularization within the tumour.

We report the case of a 7-year-old Albanian girl who was presented with abdominal pain and a palpable mass in the region of her right ovary. Initially the girl complained about abdominal pain in all quadrants of the abdomen. Later the pain was localized on the right side. According to her mother the pregnancy with her daughter was uncomplicated; the girl had never been ill before, had no allergies, and was physically healthy looking except for light skin paleness. The laboratory results were normal except for haemoglobin (11, 8 g/dL) and haematocrit (34,6%). The radiological CT scan of the abdomen and pelvis revealed a massive, solid, and clearly bordered formation with the dimensions 12 × 8 × 15 cm. The suspicious formation showed a connection to the right ovary that was hyperdense and had an inhomogeneous appearance. Some hypodense tissue within the tumour mass was identified as probably “fatty tissue” (Figures 1(a) and 1(b)). After an additional examination with intravenous contrast medium the tumour mass showed a raised vascularization within the tumor (Figures 2(a) and 2(b)).

The radiologist assumed an embryological origin of the tumour mass and suspected an “Immature teratoma of the right ovary with probable adhesion to the surrounding area.”

**Figure 3:**
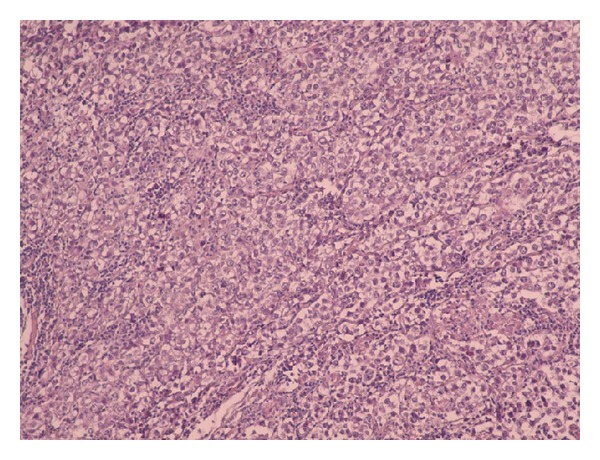
Uniform tumor cells arranged in nests, separated by delicate fibrous stroma rich in lymphocytes (×5; H&E stain).

**Figure 4:**
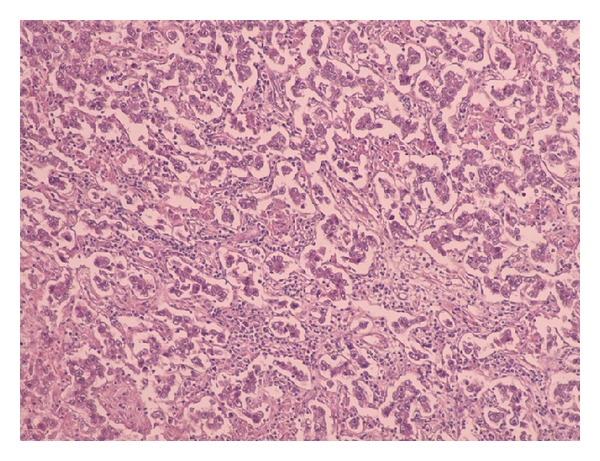
Medium-sized tumor cells with eosinophilic cytoplasm and central nuclei with vesicular chromatin (×10; H&E stain).

**Figure 5:**
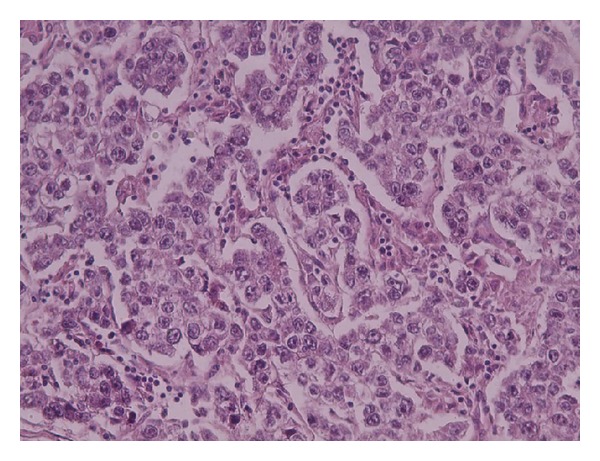
Higher magnification showing focal prominent nucleoli of the tumor cells (×20; H&E stain).

**Figure 6:**
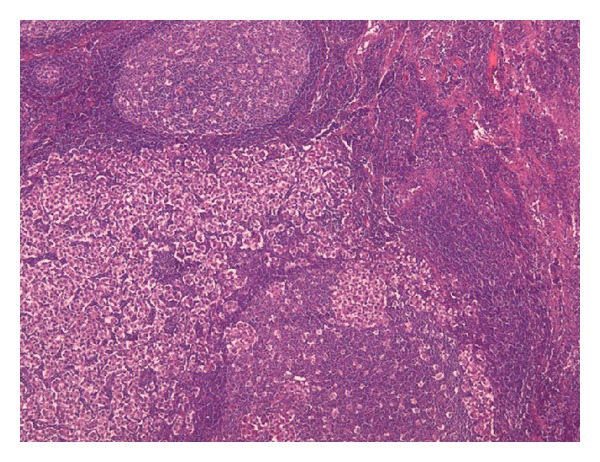
Lymph node metastasis with area of the tumor cells partially replacing the lymph node structure (×2.5; H&E stain).

The transversal infraumbilical laparotomy revealed a huge formation which involved both ovaries and the uterus. After intrasurgically consulting the gynaecologist the surgeons made the indication for a total hysterectomy with bilateral salpingo-oophorectomy. After extirpating the tumour mass suspicious lymph nodes were also removed and the surgical preparation was sent to the pathologist. The histopathological examination revealed a dysgerminoma with FIGO stage IIIc. The tumor mass was 692 g and measured 17 × 11 × 8.5 cm. On cut surface the tumor had multinodular appearance with confluent areas of necrosis and hemorrhage. Histological examination revealed infiltrating islands of uniform tumor cells with ample clear cytoplasm with central or slightly excentric nuclei with a vesicular chromatin pattern and prominent nucleoli (Figures 3 and 5). The tumor islands were separated by collagenous stroma densely infiltrated by lymphocytes (Figure 4). The tumor cells were negative for CD45 (LCA) and S-100. PLAP (placental alkaline phosphatase) was not available at our institution; however, the classical histological findings in routine stains (H&E), lack of expression of hematopoietic markers, and clinical findings were sufficient evidence of “seminoma-like” germ-cell nature of this tumor, namely, dysgerminoma. Additionally, one lymph node was positive for metastatic tumor deposits (Figure 6).

## 3. Discussion

A review of Andrés et al. revealed the percentage of dysgerminomas (15%) in solid tumours in childhood [7]. The mean age at diagnosis is 14 + 2.7 years and the majority of the patients are postpubertal [3] in contrast to our patient, who was 7 years old and did not show any signs of puberty at presentation. Vicus et al. published data of the occurrence of pure ovarian dysgerminomas: 72.3% stage I, 4.6% stage II, and 21.5% stage III disease. The initial treatment was surgery with 72.2% of the patients undergoing unilateral oophorectomy and 21.5% bilateral oophorectomy +/− hysterectomy [8]. The indication for bilateral oophorectomy and hysterectomy in our case was intrasurgically made with a gynaecologist. The reason therefore was a macroscopically visible infiltration of both ovaries and the uterus which coalesce to a huge tumour mass. Although fertility-sparing surgery is the main aim and is possible in 70% of cases [3], the indication for bilateral oophorectomy and hysterectomy in our case was obvious. We could not see any possibilities of sparing the infiltrated uterus and one ovary without putting the child into risk of a further growing of the tumour and probably leading to metastasis into other organs. Similar to our case Nishio et al. made the indication for radical surgery at stages III and IV [9]. In contrast to this Vicus et al. showed that fertility-sparing surgery in women with pure ovarian dysgerminoma led in 8 of 65 cases to 12 pregnancies and 12 live births.

Drozyńska et al. published differences of dysgerminomas in young children (younger than 10 years) and older children (between 11 and 18 years) regarding histology, primary localization, and biochemical markers. The marker AFP was higher in younger patients (76% versus 44%), whereas *β*-HCG levels were increased in older patients (40% versus 9%). Compared to these findings our case is rare regarding the age and the occurrence of a FIGO stage IIIc.

Lymph node metastasis is present in 28% of dysgerminomas and is significantly associated with poor survival [5]. In order to evaluate the prognosis and find adequate therapy options lymphadenectomy is indicated. The result of the lymphadenectomy in our case was positive and showed tumour cells within the lymph node which is according to Kumar et al. a predictor for poor survival [5].

Despite these tumours being rare an overall survival of 97% can be achieved with conservative surgery and platinum-based chemotherapy [7]. Adjuvant chemotherapy in combination with initial surgery shows promising results concerning outcome and fertility [9]. Quero-Hernández et al. used mean 4 chemotherapy cycles including cisplatin, etoposide, and bleomycin [10]. We used also 4 chemotherapy cycles (including cisplatin, etoposide, and bleomycin) and radiotherapy. She is still in complete remission approximately one year after presentation. We believe that our report on the challenges of diagnosis and treatment in this case can help clinicians to better understand and manage these pathologies.

## 4. Conclusion

Our case shows that inconspicuous symptoms like abdominal pain, abdominal distention, nausea, and vomiting might occur due to a malignant ovarian germ cell tumour. Although dysgerminomas rarely occur in childhood, the physician should not exclude the possibility of dysgerminomas appearing in this age. Therefore a complete clinical examination with radiological scans is necessary in order not to miss growing malignant formations. In cooperation with gynaecologists and paediatricians the best individual therapy option should be found. Adjuvant chemotherapy and radiotherapy show favourable outcome and future genetic examinations will hopefully reveal new biological and genetic targets to improve overall survival and fertility.

## Abbreviations

MOGT: Malignant ovarian germ cell tumor
PFS: Progression-free survival.
